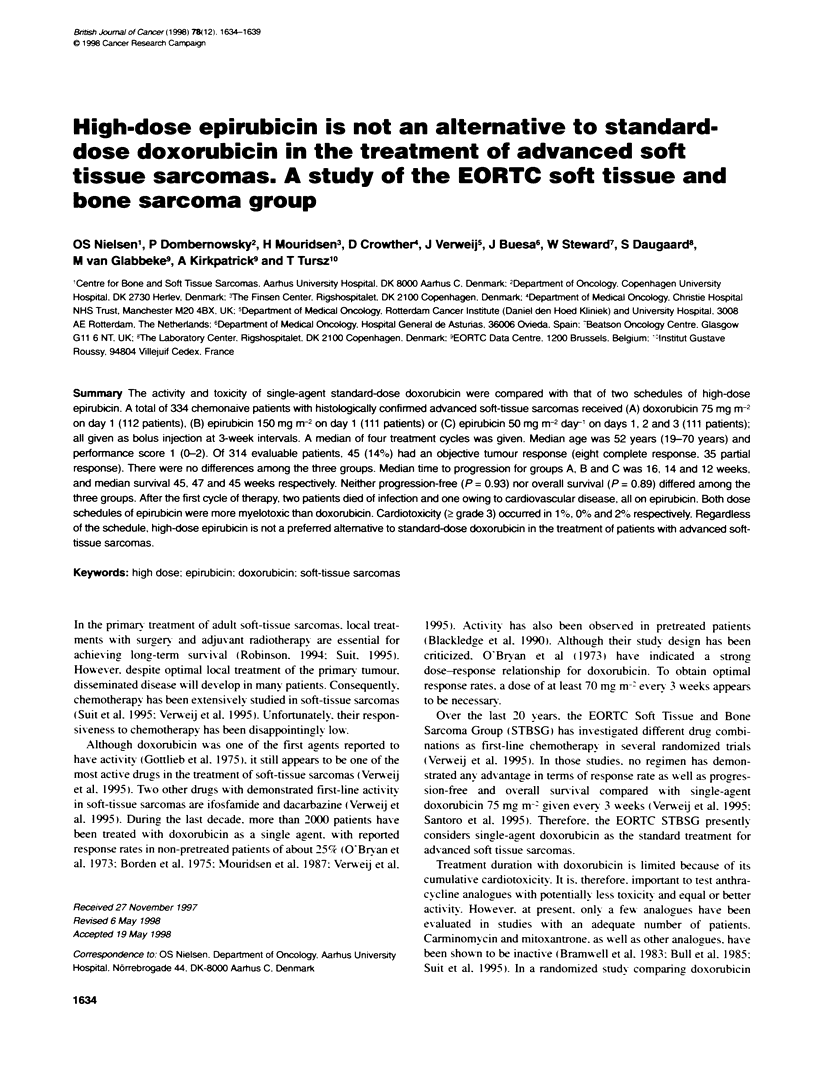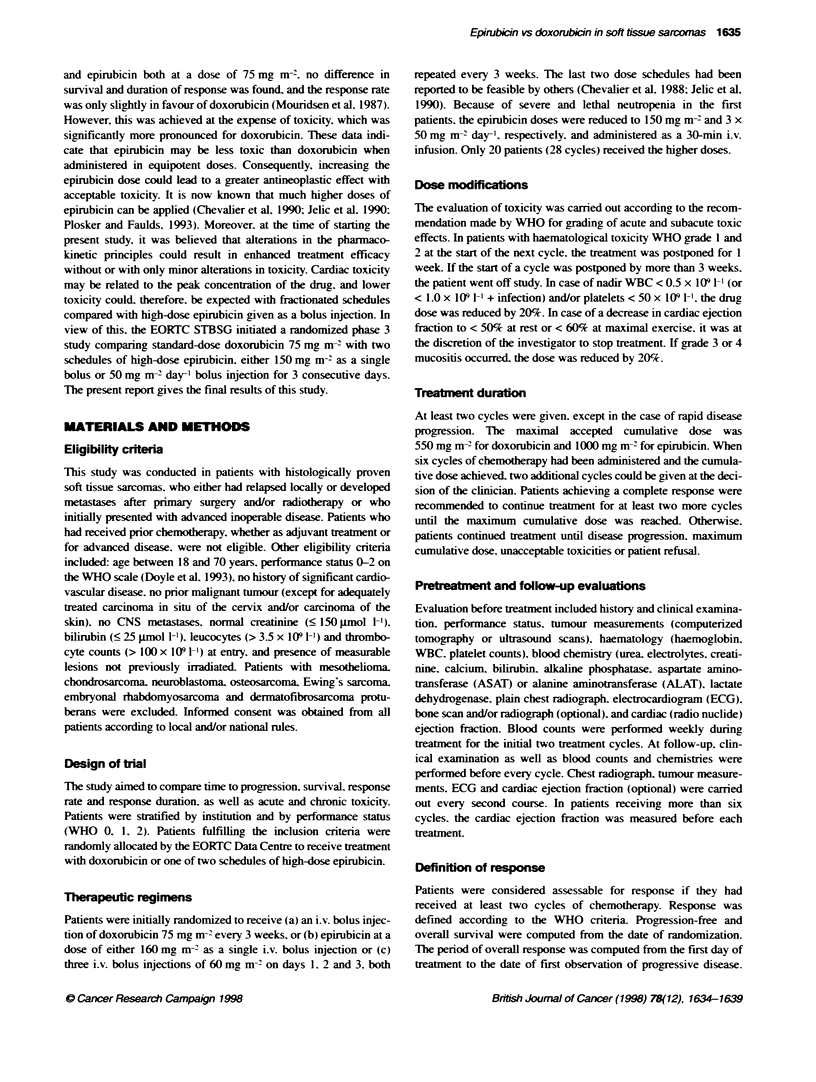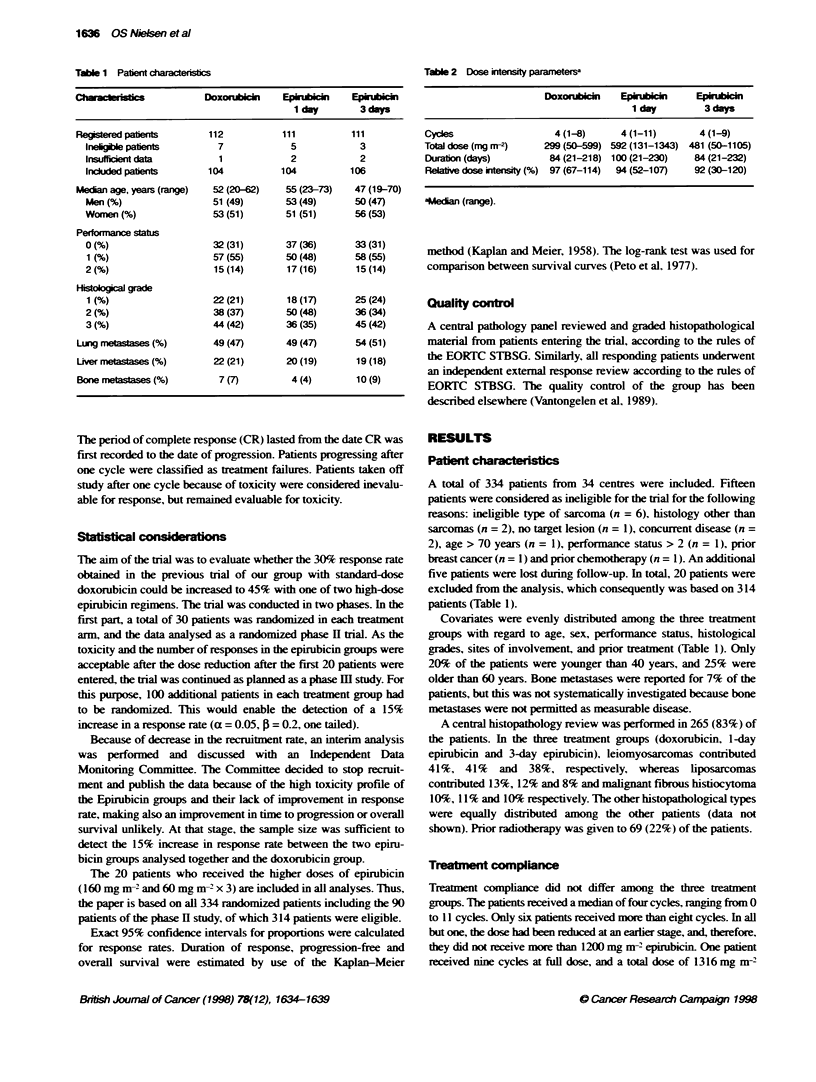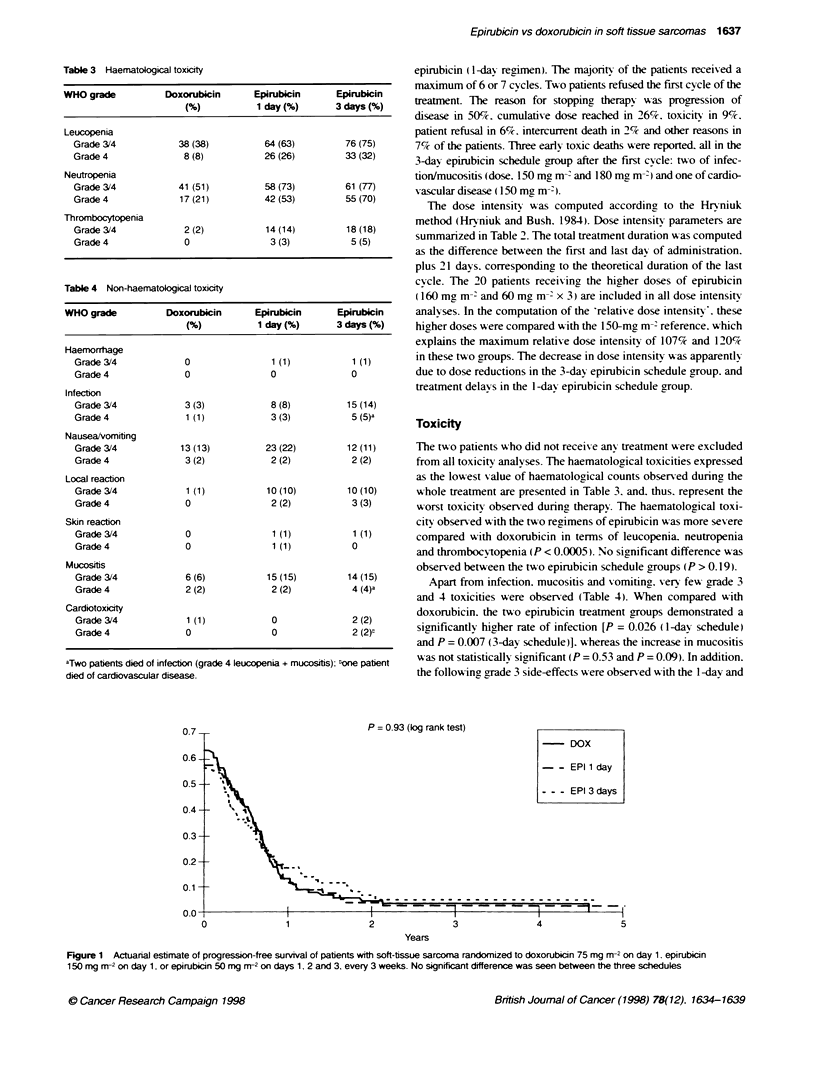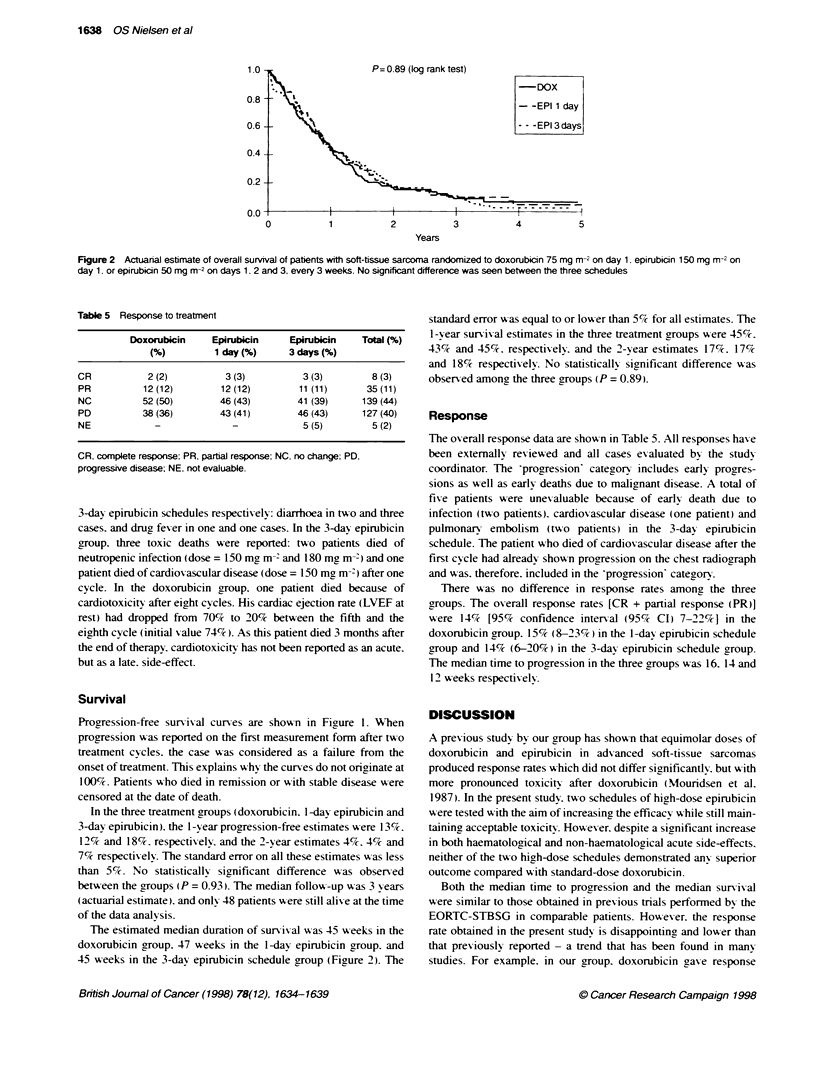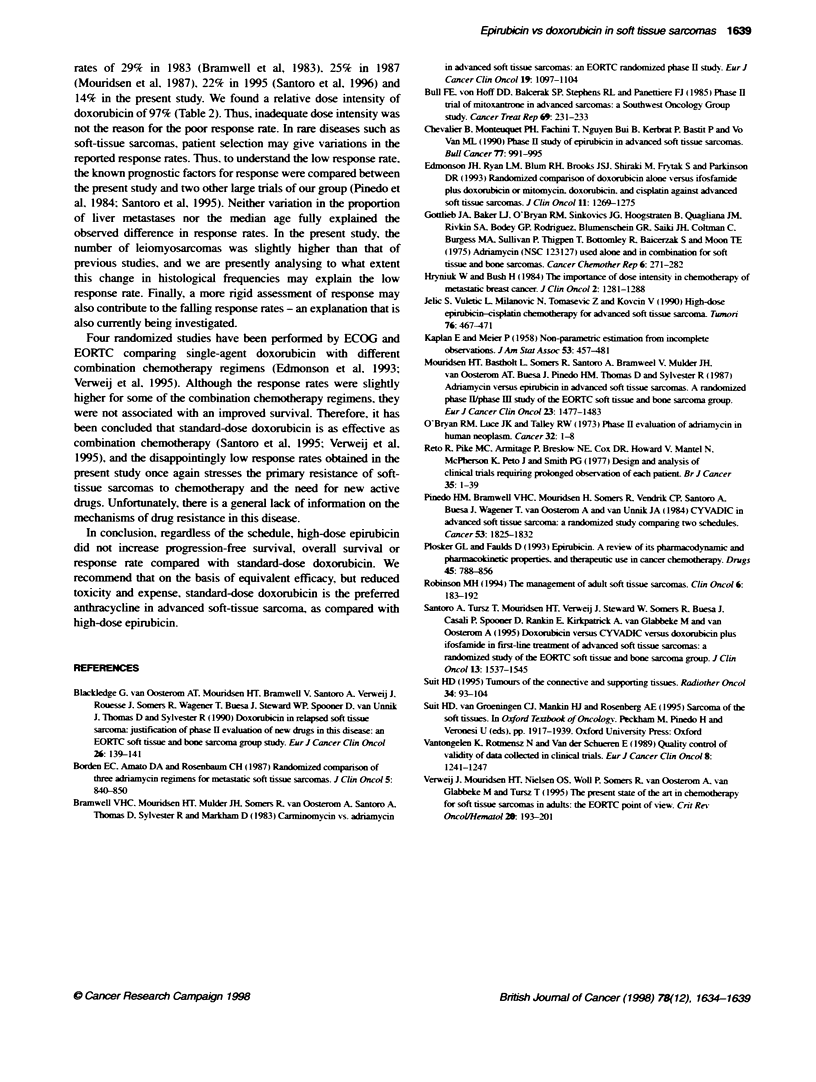# High-dose epirubicin is not an alternative to standard-dose doxorubicin in the treatment of advanced soft tissue sarcomas. A study of the EORTC soft tissue and bone sarcoma group.

**DOI:** 10.1038/bjc.1998.735

**Published:** 1998-12

**Authors:** O. S. Nielsen, P. Dombernowsky, H. Mouridsen, D. Crowther, J. Verweij, J. Buesa, W. Steward, S. Daugaard, M. van Glabbeke, A. Kirkpatrick, T. Tursz

**Affiliations:** Centre for Bone and Soft Tissue Sarcomas, Aarhus University Hospital, Denmark.

## Abstract

The activity and toxicity of single-agent standard-dose doxorubicin were compared with that of two schedules of high-dose epirubicin. A total of 334 chemonaive patients with histologically confirmed advanced soft-tissue sarcomas received (A) doxorubicin 75 mg m(-2) on day 1 (112 patients), (B) epirubicin 150 mg m(-2) on day 1 (111 patients) or (C) epirubicin 50 mg m(-2) day(-1) on days 1, 2 and 3 (111 patients); all given as bolus injection at 3-week intervals. A median of four treatment cycles was given. Median age was 52 years (19-70 years) and performance score 1 (0-2). Of 314 evaluable patients, 45 (14%) had an objective tumour response (eight complete response, 35 partial response). There were no differences among the three groups. Median time to progression for groups A, B and C was 16, 14 and 12 weeks, and median survival 45, 47 and 45 weeks respectively. Neither progression-free (P = 0.93) nor overall survival (P = 0.89) differed among the three groups. After the first cycle of therapy, two patients died of infection and one owing to cardiovascular disease, all on epirubicin. Both dose schedules of epirubicin were more myelotoxic than doxorubicin. Cardiotoxicity (> or = grade 3) occurred in 1%, 0% and 2% respectively. Regardless of the schedule, high-dose epirubicin is not a preferred alternative to standard-dose doxorubicin in the treatment of patients with advanced soft-tissue sarcomas.


British Journal of Cancer (1998) 78(12), 1634–1639
© 1998 Cancer Research Campaign

### High-dose epirubicin is not an alternative to standard-dose doxorubicin in the treatment of advanced soft tissue sarcomas. A study of the EORTC soft tissue and bone sarcoma group

**OS Nielsen[1], P Dombernowsky[2], H Mouridsen[3], D Crowther[4], J Verweij[5], J Buesa[6], W Steward[7], S Daugaard[8], M van Glabbeke[9], A Kirkpatrick[9] and T Tursz[10]**

[1]Centre for Bone and Soft Tissue Sarcomas, Aarhus University Hospital, DK 8000 Aarhus C, Denmark; [2]Department of Oncology, Copenhagen University Hospital, DK 2730 Herlev, Denmark; [3]The Finsen Center, Rigshospitalet, DK 2100 Copenhagen, Denmark; [4]Department of Medical Oncology, Christie Hospital NHS Trust, Manchester M20 4BX, UK; [5]Department of Medical Oncology, Rotterdam Cancer Institute (Daniel den Hoed Kliniek) and University Hospital, 3008 AE Rotterdam, The Netherlands; [6]Department of Medical Oncology, Hospital General de Asturias, 36006 Ovieda, Spain; [7]Beatson Oncology Centre, Glasgow G11 6 NT, UK; [8]The Laboratory Center, Rigshospitalet, DK 2100 Copenhagen, Denmark; [9]EORTC Data Centre, 1200 Brussels, Belgium; [10]Institut Gustave Roussy, 94804 Villejuif Cedex, France

**Summary** The activity and toxicity of single-agent standard-dose doxorubicin were compared with that of two schedules of high-dose epirubicin. A total of 334 chemonaive patients with histologically confirmed advanced soft-tissue sarcomas received (A) doxorubicin 75 mg $m^{-2}$ on day 1 (112 patients), (B) epirubicin 150 mg $m^{-2}$ on day 1 (111 patients) or (C) epirubicin 50 mg $m^{-2}$ $day^{-1}$ on days 1, 2 and 3 (111 patients); all given as bolus injection at 3-week intervals. A median of four treatment cycles was given. Median age was 52 years (19–70 years) and performance score 1 (0–2). Of 314 evaluable patients, 45 (14%) had an objective tumour response (eight complete response, 35 partial response). There were no differences among the three groups. Median time to progression for groups A, B and C was 16, 14 and 12 weeks, and median survival 45, 47 and 45 weeks respectively. Neither progression-free ($P = 0.93$) nor overall survival ($P = 0.89$) differed among the three groups. After the first cycle of therapy, two patients died of infection and one owing to cardiovascular disease, all on epirubicin. Both dose schedules of epirubicin were more myelotoxic than doxorubicin. Cardiotoxicity (≥ grade 3) occurred in 1%, 0% and 2% respectively. Regardless of the schedule, high-dose epirubicin is not a preferred alternative to standard-dose doxorubicin in the treatment of patients with advanced soft-tissue sarcomas.

**Keywords:** high dose; epirubicin; doxorubicin; soft-tissue sarcomas

In the primary treatment of adult soft-tissue sarcomas, local treatments with surgery and adjuvant radiotherapy are essential for achieving long-term survival (Robinson, 1994; Suit, 1995). However, despite optimal local treatment of the primary tumour, disseminated disease will develop in many patients. Consequently, chemotherapy has been extensively studied in soft-tissue sarcomas (Suit et al, 1995; Verweij et al, 1995). Unfortunately, their responsiveness to chemotherapy has been disappointingly low.

Although doxorubicin was one of the first agents reported to have activity (Gottlieb et al, 1975), it still appears to be one of the most active drugs in the treatment of soft-tissue sarcomas (Verweij et al, 1995). Two other drugs with demonstrated first-line activity in soft-tissue sarcomas are ifosfamide and dacarbazine (Verweij et al, 1995). During the last decade, more than 2000 patients have been treated with doxorubicin as a single agent, with reported response rates in non-pretreated patients of about 25% (O'Bryan et al, 1973; Borden et al, 1975; Mouridsen et al, 1987; Verweij et al, 1995). Activity has also been observed in pretreated patients (Blackledge et al, 1990). Although their study design has been criticized, O'Bryan et al (1973) have indicated a strong dose–response relationship for doxorubicin. To obtain optimal response rates, a dose of at least 70 mg $m^{-2}$ every 3 weeks appears to be necessary.

Over the last 20 years, the EORTC Soft Tissue and Bone Sarcoma Group (STBSG) has investigated different drug combinations as first-line chemotherapy in several randomized trials (Verweij et al, 1995). In those studies, no regimen has demonstrated any advantage in terms of response rate as well as progression-free and overall survival compared with single-agent doxorubicin 75 mg $m^{-2}$ given every 3 weeks (Verweij et al, 1995; Santoro et al, 1995). Therefore, the EORTC STBSG presently considers single-agent doxorubicin as the standard treatment for advanced soft tissue sarcomas.

Treatment duration with doxorubicin is limited because of its cumulative cardiotoxicity. It is, therefore, important to test anthracycline analogues with potentially less toxicity and equal or better activity. However, at present, only a few analogues have been evaluated in studies with an adequate number of patients. Carminomycin and mitoxantrone, as well as other analogues, have been shown to be inactive (Bramwell et al, 1983; Bull et al, 1985; Suit et al, 1995). In a randomized study comparing doxorubicin

*Received 27 November 1997*
*Revised 6 May 1998*
*Accepted 19 May 1998*

*Correspondence to:* OS Nielsen, Department of Oncology, Aarhus University Hospital, Nörrebrogade 44, DK-8000 Aarhus C, Denmark and epirubicin both at a dose of 75 mg $m^{-2}$, no difference in survival and duration of response was found, and the response rate was only slightly in favour of doxorubicin (Mouridsen et al, 1987). However, this was achieved at the expense of toxicity, which was significantly more pronounced for doxorubicin. These data indicate that epirubicin may be less toxic than doxorubicin when administered in equipotent doses. Consequently, increasing the epirubicin dose could lead to a greater antineoplastic effect with acceptable toxicity. It is now known that much higher doses of epirubicin can be applied (Chevalier et al, 1990; Jelic et al, 1990; Plosker and Faulds, 1993). Moreover, at the time of starting the present study, it was believed that alterations in the pharmacokinetic principles could result in enhanced treatment efficacy without or with only minor alterations in toxicity. Cardiac toxicity may be related to the peak concentration of the drug, and lower toxicity could, therefore, be expected with fractionated schedules compared with high-dose epirubicin given as a bolus injection. In view of this, the EORTC STBSG initiated a randomized phase 3 study comparing standard-dose doxorubicin 75 mg $m^{-2}$ with two schedules of high-dose epirubicin, either 150 mg $m^{-2}$ as a single bolus or 50 mg $m^{-2}$ $day^{-1}$ bolus injection for 3 consecutive days. The present report gives the final results of this study.

### MATERIALS AND METHODS

### Eligibility criteria

This study was conducted in patients with histologically proven soft tissue sarcomas, who either had relapsed locally or developed metastases after primary surgery and/or radiotherapy or who initially presented with advanced inoperable disease. Patients who had received prior chemotherapy, whether as adjuvant treatment or for advanced disease, were not eligible. Other eligibility criteria included: age between 18 and 70 years, performance status 0–2 on the WHO scale (Doyle et al, 1993), no history of significant cardiovascular disease, no prior malignant tumour (except for adequately treated carcinoma in situ of the cervix and/or carcinoma of the skin), no CNS metastases, normal creatinine ($\leq$ 150 µmol $l^{-1}$), bilirubin ($\leq$ 25 µmol $l^{-1}$), leucocytes ($> 3.5 \times 10^9$ $l^{-1}$) and thrombocyte counts ($> 100 \times 10^9$ $l^{-1}$) at entry, and presence of measurable lesions not previously irradiated. Patients with mesothelioma, chondrosarcoma, neuroblastoma, osteosarcoma, Ewing's sarcoma, embryonal rhabdomyosarcoma and dermatofibrosarcoma protuberans were excluded. Informed consent was obtained from all patients according to local and/or national rules.

### Design of trial

The study aimed to compare time to progression, survival, response rate and response duration, as well as acute and chronic toxicity. Patients were stratified by institution and by performance status (WHO 0, 1, 2). Patients fulfilling the inclusion criteria were randomly allocated by the EORTC Data Centre to receive treatment with doxorubicin or one of two schedules of high-dose epirubicin.

### Therapeutic regimens

Patients were initially randomized to receive (a) an i.v. bolus injection of doxorubicin 75 mg $m^{-2}$ every 3 weeks, or (b) epirubicin at a dose of either 160 mg $m^{-2}$ as a single i.v. bolus injection or (c) three i.v. bolus injections of 60 mg $m^{-2}$ on days 1, 2 and 3, both repeated every 3 weeks. The last two dose schedules had been reported to be feasible by others (Chevalier et al, 1988; Jelic et al, 1990). Because of severe and lethal neutropenia in the first patients, the epirubicin doses were reduced to 150 mg $m^{-2}$ and $3 \times 50$ mg $m^{-2}$ $day^{-1}$, respectively, and administered as a 30-min i.v. infusion. Only 20 patients (28 cycles) received the higher doses.

### Dose modifications

The evaluation of toxicity was carried out according to the recommendation made by WHO for grading of acute and subacute toxic effects. In patients with haematological toxicity WHO grade 1 and 2 at the start of the next cycle, the treatment was postponed for 1 week. If the start of a cycle was postponed by more than 3 weeks, the patient went off study. In case of nadir WBC $< 0.5 \times 10^9$ $l^{-1}$ (or $< 1.0 \times 10^9$ $l^{-1}$ + infection) and/or platelets $< 50 \times 10^9$ $l^{-1}$, the drug dose was reduced by 20%. In case of a decrease in cardiac ejection fraction to < 50% at rest or < 60% at maximal exercise, it was at the discretion of the investigator to stop treatment. If grade 3 or 4 mucositis occurred, the dose was reduced by 20%.

### Treatment duration

At least two cycles were given, except in the case of rapid disease progression. The maximal accepted cumulative dose was 550 mg $m^{-2}$ for doxorubicin and 1000 mg $m^{-2}$ for epirubicin. When six cycles of chemotherapy had been administered and the cumulative dose achieved, two additional cycles could be given at the decision of the clinician. Patients achieving a complete response were recommended to continue treatment for at least two more cycles until the maximum cumulative dose was reached. Otherwise, patients continued treatment until disease progression, maximum cumulative dose, unacceptable toxicities or patient refusal.

### Pretreatment and follow-up evaluations

Evaluation before treatment included history and clinical examination, performance status, tumour measurements (computerized tomography or ultrasound scans), haematology (haemoglobin, WBC, platelet counts), blood chemistry (urea, electrolytes, creatinine, calcium, bilirubin, alkaline phosphatase, aspartate aminotransferase (ASAT) or alanine aminotransferase (ALAT), lactate dehydrogenase, plain chest radiograph, electrocardiogram (ECG), bone scan and/or radiograph (optional), and cardiac (radio nuclide) ejection fraction. Blood counts were performed weekly during treatment for the initial two treatment cycles. At follow-up, clinical examination as well as blood counts and chemistries were performed before every cycle. Chest radiograph, tumour measurements, ECG and cardiac ejection fraction (optional) were carried out every second course. In patients receiving more than six cycles, the cardiac ejection fraction was measured before each treatment.

### Definition of response

Patients were considered assessable for response if they had received at least two cycles of chemotherapy. Response was defined according to the WHO criteria. Progression-free and overall survival were computed from the date of randomization. The period of overall response was computed from the first day of treatment to the date of first observation of progressive disease.

© Cancer Research Campaign 1998

Table 1 Patient characteristics

| Characteristics | Doxorubicin | Epirubicin 1 day | Epirubicin 3 days |
|---|---|---|---|
| Registered patients | 112 | 111 | 111 |
| Ineligible patients | 7 | 5 | 3 |
| Insufficient data | 1 | 2 | 2 |
| Included patients | 104 | 104 | 106 |
| Median age, years (range) | 52 (20–62) | 55 (23–73) | 47 (19–70) |
| Men (%) | 51 (49) | 53 (49) | 50 (47) |
| Women (%) | 53 (51) | 51 (51) | 56 (53) |
| Performance status | | | |
| 0 (%) | 32 (31) | 37 (36) | 33 (31) |
| 1 (%) | 57 (55) | 50 (48) | 58 (55) |
| 2 (%) | 15 (14) | 17 (16) | 15 (14) |
| Histological grade | | | |
| 1 (%) | 22 (21) | 18 (17) | 25 (24) |
| 2 (%) | 38 (37) | 50 (48) | 36 (34) |
| 3 (%) | 44 (42) | 36 (35) | 45 (42) |
| Lung metastases (%) | 49 (47) | 49 (47) | 54 (51) |
| Liver metastases (%) | 22 (21) | 20 (19) | 19 (18) |
| Bone metastases (%) | 7 (7) | 4 (4) | 10 (9) |

The period of complete response (CR) lasted from the date CR was first recorded to the date of progression. Patients progressing after one cycle were classified as treatment failures. Patients taken off study after one cycle because of toxicity were considered inevaluable for response, but remained evaluable for toxicity.

### Statistical considerations

The aim of the trial was to evaluate whether the 30% response rate obtained in the previous trial of our group with standard-dose doxorubicin could be increased to 45% with one of two high-dose epirubicin regimens. The trial was conducted in two phases. In the first part, a total of 30 patients was randomized in each treatment arm, and the data analysed as a randomized phase II trial. As the toxicity and the number of responses in the epirubicin groups were acceptable after the dose reduction after the first 20 patients were entered, the trial was continued as planned as a phase III study. For this purpose, 100 additional patients in each treatment group had to be randomized. This would enable the detection of a 15% increase in a response rate ($\alpha = 0.05$, $\beta = 0.2$, one tailed).

Because of decrease in the recruitment rate, an interim analysis was performed and discussed with an Independent Data Monitoring Committee. The Committee decided to stop recruitment and publish the data because of the high toxicity profile of the Epirubicin groups and their lack of improvement in response rate, making also an improvement in time to progression or overall survival unlikely. At that stage, the sample size was sufficient to detect the 15% increase in response rate between the two epirubicin groups analysed together and the doxorubicin group.

The 20 patients who received the higher doses of epirubicin (160 mg $m^{-2}$ and 60 mg $m^{-2} \times 3$) are included in all analyses. Thus, the paper is based on all 334 randomized patients including the 90 patients of the phase II study, of which 314 patients were eligible.

Exact 95% confidence intervals for proportions were calculated for response rates. Duration of response, progression-free and overall survival were estimated by use of the Kaplan–Meier method (Kaplan and Meier, 1958). The log-rank test was used for comparison between survival curves (Peto et al, 1977).

Table 2 Dose intensity parameters[a]

| | Doxorubicin | Epirubicin 1 day | Epirubicin 3 days |
|---|---|---|---|
| Cycles | 4 (1–8) | 4 (1–11) | 4 (1–9) |
| Total dose (mg $m^{-2}$) | 299 (50–599) | 592 (131–1343) | 481 (50–1105) |
| Duration (days) | 84 (21–218) | 100 (21–230) | 84 (21–232) |
| Relative dose intensity (%) | 97 (67–114) | 94 (52–107) | 92 (30–120) |

[a]Median (range).

### Quality control

A central pathology panel reviewed and graded histopathological material from patients entering the trial, according to the rules of the EORTC STBSG. Similarly, all responding patients underwent an independent external response review according to the rules of EORTC STBSG. The quality control of the group has been described elsewhere (Vantongelen et al, 1989).

### RESULTS

### Patient characteristics

A total of 334 patients from 34 centres were included. Fifteen patients were considered as ineligible for the trial for the following reasons: ineligible type of sarcoma ($n = 6$), histology other than sarcomas ($n = 2$), no target lesion ($n = 1$), concurrent disease ($n = 2$), age > 70 years ($n = 1$), performance status > 2 ($n = 1$), prior breast cancer ($n = 1$) and prior chemotherapy ($n = 1$). An additional five patients were lost during follow-up. In total, 20 patients were excluded from the analysis, which consequently was based on 314 patients (Table 1).

Covariates were evenly distributed among the three treatment groups with regard to age, sex, performance status, histological grades, sites of involvement, and prior treatment (Table 1). Only 20% of the patients were younger than 40 years, and 25% were older than 60 years. Bone metastases were reported for 7% of the patients, but this was not systematically investigated because bone metastases were not permitted as measurable disease.

A central histopathology review was performed in 265 (83%) of the patients. In the three treatment groups (doxorubicin, 1-day epirubicin and 3-day epirubicin), leiomyosarcomas contributed 41%, 41% and 38%, respectively, whereas liposarcomas contributed 13%, 12% and 8% and malignant fibrous histiocytoma 10%, 11% and 10% respectively. The other histopathological types were equally distributed among the other patients (data not shown). Prior radiotherapy was given to 69 (22%) of the patients.

### Treatment compliance

Treatment compliance did not differ among the three treatment groups. The patients received a median of four cycles, ranging from 0 to 11 cycles. Only six patients received more than eight cycles. In all but one, the dose had been reduced at an earlier stage, and, therefore, they did not receive more than 1200 mg $m^{-2}$ epirubicin. One patient received nine cycles at full dose, and a total dose of 1316 mg $m^{-2}$

© Cancer Research Campaign 1998

**Table 3** Haematological toxicity

| WHO grade | Doxorubicin (%) | Epirubicin 1 day (%) | Epirubicin 3 days (%) |
|---|---|---|---|
| Leucopenia | | | |
| Grade 3/4 | 38 (38) | 64 (63) | 76 (75) |
| Grade 4 | 8 (8) | 26 (26) | 33 (32) |
| Neutropenia | | | |
| Grade 3/4 | 41 (51) | 58 (73) | 61 (77) |
| Grade 4 | 17 (21) | 42 (53) | 55 (70) |
| Thrombocytopenia | | | |
| Grade 3/4 | 2 (2) | 14 (14) | 18 (18) |
| Grade 4 | 0 | 3 (3) | 5 (5) |

**Table 4** Non-haematological toxicity

| WHO grade | Doxorubicin (%) | Epirubicin 1 day (%) | Epirubicin 3 days (%) |
|---|---|---|---|
| Haemorrhage | | | |
| Grade 3/4 | 0 | 1 (1) | 1 (1) |
| Grade 4 | 0 | 0 | 0 |
| Infection | | | |
| Grade 3/4 | 3 (3) | 8 (8) | 15 (14) |
| Grade 4 | 1 (1) | 3 (3) | 5 (5)[a] |
| Nausea/vomiting | | | |
| Grade 3/4 | 13 (13) | 23 (22) | 12 (11) |
| Grade 4 | 3 (2) | 2 (2) | 2 (2) |
| Local reaction | | | |
| Grade 3/4 | 1 (1) | 10 (10) | 10 (10) |
| Grade 4 | 0 | 2 (2) | 3 (3) |
| Skin reaction | | | |
| Grade 3/4 | 0 | 1 (1) | 1 (1) |
| Grade 4 | 0 | 1 (1) | 0 |
| Mucositis | | | |
| Grade 3/4 | 6 (6) | 15 (15) | 14 (15) |
| Grade 4 | 2 (2) | 2 (2) | 4 (4)[a] |
| Cardiotoxicity | | | |
| Grade 3/4 | 1 (1) | 0 | 2 (2) |
| Grade 4 | 0 | 0 | 2 (2)[b] |

[a]Two patients died of infection (grade 4 leucopenia + mucositis): [b]one patient died of cardiovascular disease.

epirubicin (1-day regimen). The majority of the patients received a maximum of 6 or 7 cycles. Two patients refused the first cycle of the treatment. The reason for stopping therapy was progression of disease in 50%, cumulative dose reached in 26%, toxicity in 9%, patient refusal in 6%, intercurrent death in 2% and other reasons in 7% of the patients. Three early toxic deaths were reported, all in the 3-day epirubicin schedule group after the first cycle: two of infection/mucositis (dose, 150 mg $m^{-2}$ and 180 mg $m^{-2}$) and one of cardiovascular disease (150 mg $m^{-2}$).

The dose intensity was computed according to the Hryniuk method (Hryniuk and Bush, 1984). Dose intensity parameters are summarized in Table 2. The total treatment duration was computed as the difference between the first and last day of administration, plus 21 days, corresponding to the theoretical duration of the last cycle. The 20 patients receiving the higher doses of epirubicin (160 mg $m^{-2}$ and 60 mg $m^{-2}$ × 3) are included in all dose intensity analyses. In the computation of the 'relative dose intensity', these higher doses were compared with the 150-mg $m^{-2}$ reference, which explains the maximum relative dose intensity of 107% and 120% in these two groups. The decrease in dose intensity was apparently due to dose reductions in the 3-day epirubicin schedule group, and treatment delays in the 1-day epirubicin schedule group.

### Toxicity

The two patients who did not receive any treatment were excluded from all toxicity analyses. The haematological toxicities expressed as the lowest value of haematological counts observed during the whole treatment are presented in Table 3, and, thus, represent the worst toxicity observed during therapy. The haematological toxicity observed with the two regimens of epirubicin was more severe compared with doxorubicin in terms of leucopenia, neutropenia and thrombocytopenia ($P < 0.0005$). No significant difference was observed between the two epirubicin schedule groups ($P > 0.19$).

Apart from infection, mucositis and vomiting, very few grade 3 and 4 toxicities were observed (Table 4). When compared with doxorubicin, the two epirubicin treatment groups demonstrated a significantly higher rate of infection [$P = 0.026$ (1-day schedule) and $P = 0.007$ (3-day schedule)], whereas the increase in mucositis was not statistically significant ($P = 0.53$ and $P = 0.09$). In addition, the following grade 3 side-effects were observed with the 1-day and

**Figure 1** Actuarial estimate of progression-free survival of patients with soft-tissue sarcoma randomized to doxorubicin 75 mg $m^{-2}$ on day 1, epirubicin 150 mg $m^{-2}$ on day 1, or epirubicin 50 mg $m^{-2}$ on days 1, 2 and 3, every 3 weeks. No significant difference was seen between the three schedules

**Figure 2** Actuarial estimate of overall survival of patients with soft-tissue sarcoma randomized to doxorubicin 75 mg $m^{-2}$ on day 1, epirubicin 150 mg $m^{-2}$ on day 1, or epirubicin 50 mg $m^{-2}$ on days 1, 2 and 3, every 3 weeks. No significant difference was seen between the three schedules

**Table 5** Response to treatment

| | Doxorubicin (%) | Epirubicin 1 day (%) | Epirubicin 3 days (%) | Total (%) |
|---|---|---|---|---|
| CR | 2 (2) | 3 (3) | 3 (3) | 8 (3) |
| PR | 12 (12) | 12 (12) | 11 (11) | 35 (11) |
| NC | 52 (50) | 46 (43) | 41 (39) | 139 (44) |
| PD | 38 (36) | 43 (41) | 46 (43) | 127 (40) |
| NE | – | – | 5 (5) | 5 (2) |

CR, complete response; PR, partial response; NC, no change; PD, progressive disease; NE, not evaluable.

3-day epirubicin schedules respectively: diarrhoea in two and three cases, and drug fever in one and one cases. In the 3-day epirubicin group, three toxic deaths were reported: two patients died of neutropenic infection (dose = 150 mg $m^{-2}$ and 180 mg $m^{-2}$) and one patient died of cardiovascular disease (dose = 150 mg $m^{-2}$) after one cycle. In the doxorubicin group, one patient died because of cardiotoxicity after eight cycles. His cardiac ejection rate (LVEF at rest) had dropped from 70% to 20% between the fifth and the eighth cycle (initial value 74%). As this patient died 3 months after the end of therapy, cardiotoxicity has not been reported as an acute, but as a late, side-effect.

### Survival

Progression-free survival curves are shown in Figure 1. When progression was reported on the first measurement form after two treatment cycles, the case was considered as a failure from the onset of treatment. This explains why the curves do not originate at 100%. Patients who died in remission or with stable disease were censored at the date of death.

In the three treatment groups (doxorubicin, 1-day epirubicin and 3-day epirubicin), the 1-year progression-free estimates were 13%, 12% and 18%, respectively, and the 2-year estimates 4%, 4% and 7% respectively. The standard error on all these estimates was less than 5%. No statistically significant difference was observed between the groups ($P = 0.93$). The median follow-up was 3 years (actuarial estimate), and only 48 patients were still alive at the time of the data analysis.

The estimated median duration of survival was 45 weeks in the doxorubicin group, 47 weeks in the 1-day epirubicin group, and 45 weeks in the 3-day epirubicin schedule group (Figure 2). The standard error was equal to or lower than 5% for all estimates. The 1-year survival estimates in the three treatment groups were 45%, 43% and 45%, respectively, and the 2-year estimates 17%, 17% and 18% respectively. No statistically significant difference was observed among the three groups ($P = 0.89$).

### Response

The overall response data are shown in Table 5. All responses have been externally reviewed and all cases evaluated by the study coordinator. The 'progression' category includes early progressions as well as early deaths due to malignant disease. A total of five patients were unevaluable because of early death due to infection (two patients), cardiovascular disease (one patient) and pulmonary embolism (two patients) in the 3-day epirubicin schedule. The patient who died of cardiovascular disease after the first cycle had already shown progression on the chest radiograph and was, therefore, included in the 'progression' category.

There was no difference in response rates among the three groups. The overall response rates [CR + partial response (PR)] were 14% [95% confidence interval (95% CI) 7–22%] in the doxorubicin group, 15% (8–23%) in the 1-day epirubicin schedule group and 14% (6–20%) in the 3-day epirubicin schedule group. The median time to progression in the three groups was 16, 14 and 12 weeks respectively.

### DISCUSSION

A previous study by our group has shown that equimolar doses of doxorubicin and epirubicin in advanced soft-tissue sarcomas produced response rates which did not differ significantly, but with more pronounced toxicity after doxorubicin (Mouridsen et al, 1987). In the present study, two schedules of high-dose epirubicin were tested with the aim of increasing the efficacy while still maintaining acceptable toxicity. However, despite a significant increase in both haematological and non-haematological acute side-effects, neither of the two high-dose schedules demonstrated any superior outcome compared with standard-dose doxorubicin.

Both the median time to progression and the median survival were similar to those obtained in previous trials performed by the EORTC-STBSG in comparable patients. However, the response rate obtained in the present study is disappointing and lower than that previously reported – a trend that has been found in many studies. For example, in our group, doxorubicin gave response

© Cancer Research Campaign 1998

rates of 29% in 1983 (Bramwell et al, 1983), 25% in 1987 (Mouridsen et al, 1987), 22% in 1995 (Santoro et al, 1996) and 14% in the present study. We found a relative dose intensity of doxorubicin of 97% (Table 2). Thus, inadequate dose intensity was not the reason for the poor response rate. In rare diseases such as soft-tissue sarcomas, patient selection may give variations in the reported response rates. Thus, to understand the low response rate, the known prognostic factors for response were compared between the present study and two other large trials of our group (Pinedo et al, 1984; Santoro et al, 1995). Neither variation in the proportion of liver metastases nor the median age fully explained the observed difference in response rates. In the present study, the number of leiomyosarcomas was slightly higher than that of previous studies, and we are presently analysing to what extent this change in histological frequencies may explain the low response rate. Finally, a more rigid assessment of response may also contribute to the falling response rates – an explanation that is also currently being investigated.

Four randomized studies have been performed by ECOG and EORTC comparing single-agent doxorubicin with different combination chemotherapy regimens (Edmonson et al, 1993; Verweij et al, 1995). Although the response rates were slightly higher for some of the combination chemotherapy regimens, they were not associated with an improved survival. Therefore, it has been concluded that standard-dose doxorubicin is as effective as combination chemotherapy (Santoro et al, 1995; Verweij et al, 1995), and the disappointingly low response rates obtained in the present study once again stresses the primary resistance of soft-tissue sarcomas to chemotherapy and the need for new active drugs. Unfortunately, there is a general lack of information on the mechanisms of drug resistance in this disease.

In conclusion, regardless of the schedule, high-dose epirubicin did not increase progression-free survival, overall survival or response rate compared with standard-dose doxorubicin. We recommend that on the basis of equivalent efficacy, but reduced toxicity and expense, standard-dose doxorubicin is the preferred anthracycline in advanced soft-tissue sarcoma, as compared with high-dose epirubicin.

### REFERENCES

Blackledge G, van Oosterom AT, Mouridsen HT, Bramwell V, Santoro A, Verweij J, Rouesse J, Somers R, Wagener T, Buesa J, Steward WP, Spooner D, van Unnik J, Thomas D and Sylvester R (1990) Doxorubicin in relapsed soft tissue sarcoma: justification of phase II evaluation of new drugs in this disease: an EORTC soft tissue and bone sarcoma group study. *Eur J Cancer Clin Oncol* **26**: 139–141

Borden EC, Amato DA and Rosenbaum CH (1987) Randomized comparison of three adriamycin regimens for metastatic soft tissue sarcomas. *J Clin Oncol* **5**: 840–850

Bramwell VHC, Mouridsen HT, Mulder JH, Somers R, van Oosterom A, Santoro A, Thomas D, Sylvester R and Markham D (1983) Carminomycin vs. adriamycin in advanced soft tissue sarcomas: an EORTC randomized phase II study. *Eur J Cancer Clin Oncol* **19**: 1097–1104

Bull FE, von Hoff DD, Balcerak SP, Stephens RL and Panettiere FJ (1985) Phase II trial of mitoxantrone in advanced sarcomas: a Southwest Oncology Group study. *Cancer Treat Rep* **69**: 231–233

Chevalier B, Monteuquet PH, Fachini T, Nguyen Bui B, Kerbrat P, Bastit P and Vo Van ML (1990) Phase II study of epirubicin in advanced soft tissue sarcomas. *Bull Cancer* **77**: 991–995

Edmonson JH, Ryan LM, Blum RH, Brooks JSJ, Shiraki M, Frytak S and Parkinson DR (1993) Randomized comparison of doxorubicin alone versus ifosfamide plus doxorubicin or mitomycin, doxorubicin, and cisplatin against advanced soft tissue sarcomas. *J Clin Oncol* **11**: 1269–1275

Gottlieb JA, Baker LJ, O'Bryan RM, Sinkovics JG, Hoogstraten B, Quagliana JM, Rivkin SA, Bodey GP, Rodriguez, Blumenschein GR, Saiki JH, Coltman C, Burgess MA, Sullivan P, Thigpen T, Bottomley R, Baicerzak S and Moon TE (1975) Adriamycin (NSC 123127) used alone and in combination for soft tissue and bone sarcomas. *Cancer Chemother Rep* **6**: 271–282

Hryniuk W and Bush H (1984) The importance of dose intensity in chemotherapy of metastatic breast cancer. *J Clin Oncol* **2**: 1281–1288

Jelic S, Vuletic L, Milanovic N, Tomasevic Z and Kovcin V (1990) High-dose epirubicin–cisplatin chemotherapy for advanced soft tissue sarcoma. *Tumori* **76**: 467–471

Kaplan E and Meier P (1958) Non-parametric estimation from incomplete observations. *J Am Stat Assoc* **53**: 457–481

Mouridsen HT, Bastholt L, Somers R, Santoro A, Bramweel V, Mulder JH, van Oosterom AT, Buesa J, Pinedo HM, Thomas D and Sylvester R (1987) Adriamycin versus epirubicin in advanced soft tissue sarcomas. A randomized phase II/phase III study of the EORTC soft tissue and bone sarcoma group. *Eur J Cancer Clin Oncol* **23**: 1477–1483

O'Bryan RM, Luce JK and Talley RW (1973) Phase II evaluation of adriamycin in human neoplasm. *Cancer* **32**: 1–8

Reto R, Pike MC, Armitage P, Breslow NE, Cox DR, Howard V, Mantel N, McPherson K, Peto J and Smith PG (1977) Design and analysis of clinical trials requiring prolonged observation of each patient. *Br J Cancer* **35**: 1–39

Pinedo HM, Bramwell VHC, Mouridsen H, Somers R, Vendrik CP, Santoro A, Buesa J, Wagener T, van Oosterom A and van Unnik JA (1984) CYVADIC in advanced soft tissue sarcoma: a randomized study comparing two schedules. *Cancer* **53**: 1825–1832

Plosker GL and Faulds D (1993) Epirubicin. A review of its pharmacodynamic and pharmacokinetic properties, and therapeutic use in cancer chemotherapy. *Drugs* **45**: 788–856

Robinson MH (1994) The management of adult soft tissue sarcomas. *Clin Oncol* **6**: 183–192

Santoro A, Tursz T, Mouridsen HT, Verweij J, Steward W, Somers R, Buesa J, Casali P, Spooner D, Rankin E, Kirkpatrick A, van Glabbeke M and van Oosterom A (1995) Doxorubicin versus CYVADIC versus doxorubicin plus ifosfamide in first-line treatment of advanced soft tissue sarcomas: a randomized study of the EORTC soft tissue and bone sarcoma group. *J Clin Oncol* **13**: 1537–1545

Suit HD (1995) Tumours of the connective and supporting tissues. *Radiother Oncol* **34**: 93–104

Suit HD, van Groeningen CJ, Mankin HJ and Rosenberg AE (1995) Sarcoma of the soft tissues. In *Oxford Textbook of Oncology*. Peckham M, Pinedo H and Veronesi U (eds), pp. 1917–1939. Oxford University Press: Oxford

Vantongelen K, Rotmensz N and Van der Schueren E (1989) Quality control of validity of data collected in clinical trials. *Eur J Cancer Clin Oncol* **8**: 1241–1247

Verweij J, Mouridsen HT, Nielsen OS, Woll P, Somers R, van Oosterom A, van Glabbeke M and Tursz T (1995) The present state of the art in chemotherapy for soft tissue sarcomas in adults: the EORTC point of view. *Crit Rev Oncol/Hematol* **20**: 193–201

© Cancer Research Campaign 1998